# Aspirin as adjuvant therapy in childhood cancer?

**DOI:** 10.1038/bjc.2011.467

**Published:** 2011-11-10

**Authors:** G Morgan

**Affiliations:** 1 Department of Primary Care and Public Health, Cardiff University, Cardiff, UK

**Sir,**

The mini review on aspirin and cancer questions whether the medicine has been overlooked as an adjuvant therapy (Langley *et al*, 2011). Although the focus of the mini review was on adult cancer, perhaps aspirin might also be helpful in childhood cancer. There is evidence that childhood cancer survivors have increased risks of both cancer and cardiovascular disease when compared with the general population (Mulrooney *et al*, 2009; Reulen *et al*, 2011). Given the evidence supporting aspirin reducing the risk of both cancer and cardiovascular disease (Rose *et al*, 2011), potentially the medicine could be helpful in the proactive health care of survivors. Such potential does need to take into account the undesirable effects of aspirin.

Aspirin is not recommended for children, hence compared with adults, it is more difficult to make a case for aspirin as adjuvant therapy in childhood cancer. This does not mean it should not be considered at all, and perhaps the ethical aspects of low doses of aspirin as adjuvant therapy in childhood cancer need further exploration. In the meantime, perhaps a readily available alternative might be also considered. Salicylate is the putative active component of aspirin against cancer cells through several mechanisms and it might be ingested through diet. Perhaps a diet rich in salicylate, such as fruits and vegetables, could be considered as adjuvant therapy. This might also help promote the general health and wellbeing of children with cancer.
